# Cyanobacterial Sfp-type phosphopantetheinyl transferases functionalize carrier proteins of diverse biosynthetic pathways

**DOI:** 10.1038/s41598-017-12244-3

**Published:** 2017-09-19

**Authors:** Guang Yang, Yi Zhang, Nicholas K. Lee, Monica A. Cozad, Sara E. Kearney, Hendrik Luesch, Yousong Ding

**Affiliations:** 1 Department of Medicinal Chemistry and Center for Natural Products, Drug Discovery and Development, University of Florida, Gainesville, FL 32610 USA; 2 NIH Chemical Genomics Center, National Center for Advancing Translational Sciences, National Institutes of Health, Rockville, MD 20850 USA

## Abstract

Cyanobacteria produce structurally and functionally diverse polyketides, nonribosomal peptides and their hybrids. Sfp-type phosphopantetheinyl transferases (PPTases) are essential to the production of these compounds via functionalizing carrier proteins (CPs) of biosynthetic megaenzymes. However, cyanobacterial Sfp-type PPTases remain poorly characterized, posing a significant barrier to the exploitation of cyanobacteria for biotechnological and biomedical applications. Herein, we describe the detailed characterization of multiple cyanobacterial Sfp-type PPTases that were rationally selected. Biochemical characterization of these enzymes along with the prototypic enzyme Sfp from *Bacillus subtilis* demonstrated their varying specificities toward 11 recombinant CPs of different types of biosynthetic pathways from cyanobacterial and *Streptomyces* strains. Kinetic analysis further indicated that PPTases possess the higher binding affinity and catalytic efficiency toward their cognate CPs in comparison with noncognate substrates. Moreover, when chromosomally replacing the native *PPTase* gene of *Synechocystis* sp. PCC6803, two selected cyanobacterial PPTases and Sfp supported the growth of resulted mutants. Cell lysates of the cyanobacterial mutants further functionalized recombinant CP substrates. Collectively, these studies reveal the versatile catalysis of selected cyanobacterial PPTases and provide new tools to synthesize cyanobacterial natural products using *in vitro* and *in vivo* synthetic biology approaches.

## Introduction

More than 1,000 structurally diverse natural products have been isolated from cyanobacterial species over the past decades^1,2^. These compounds possess a wide array of bioactivities, e.g., antibacterial, antifungal, antiviral, immunomodulatory, protease inhibitory, and cytotoxic activities^3–5^. Evidently, monomethyl auristatin E, a derivative of cyanobacterial nonribosomal peptide/polyketide (NRP/PK) hybrid dolastatin 10^6,7^, is a clinically valuable anti-mitotic agent. Remarkably, bioinformatic analysis of over 140 cyanobacterial genomes available in the NCBI database reveals at least three NRP synthetases (NRPSs) per genome on average, while some genomes contain more than forty natural product gene clusters^8,9^. These results illustrate the untapped potential of cyanobacteria as a source of new chemicals. However, this potential remains unachievable unless capable tools are available to translate cyanobacterial genetic information into compounds^10,11^. Indeed, only a few cyanobacterial natural products including several ribosomally synthesized and post-translationally modified peptides^12,13^, barbamide (a PK/NRP hybrid) and lyngbyatoxin A (an NRP) have so far been heterologously produced in *E. coli* or *Streptomyces* sp.^14–16^. Recently, lyngbyatoxin A was also produced in the model cyanobacterium *Anabaena* sp. PCC7120, achieving a yield similar to its native producer *Moorea producens* ^17^.

Phosphopantetheinyl transferases (PPTases) play an essential role in the biosynthesis of fatty acids (FAs), NRPs, and PKs by functionalizing carrier protein (CPs) of their corresponding biosynthetic enzymes. They use coenzyme A (CoA) as one substrate to posttranslationally modify one conserved serine residue of the CPs with a 4′-phosphopantetheine (PPant) moiety, thereby converting inactive apo-form CPs to active holo-form proteins (Fig. S1)^18^. The CPs are responsible for shuttling biosynthetic intermediates among multiple catalytic domains within a module and delivering the substrate to the next module via the PPant arm. Based on sequence simila-rity and substrate specificity, PPTases are categorized into three subfamilies^19^. AcpS from *E. coli* is the prototype of the first subfamily^20^, whose members generally comprise about 120 amino acids and functionalize only the CPs of FA synthases (FASs). The second subfamily of PPTases comprises integrated domains within the type I yeast and fungal FASs, where they specifically modify the cognate CPs^19^. The last subfamily is known as Sfp-type PPTases, named after the archetypal enzyme Sfp from *Bacillus subtilis* ^21^. Compared to AcpSs, these enzymes are two times longer and functionalize a broad range of substrates including noncognate CPs for the biosynthesis of FAs, NRPs, PKs, and their hybrids^21^. Sfp-type PPTases can further be subdivided into the NRPS-preferred F/KES and PKS-favored W/KEA groups based on their sequences^22^. Given their attractive substrate promiscuity, Sfp-type PPTases have demonstrated many biotechnological applications, including site-specific protein labeling, cell imaging, and the heterologous production of microbial secondary metabolites^23–25^.

Despite the attractive potential of cyanobacterial natural products, only three PPTases from cyanobacteria have so far been biochemically characterized^17,26,27^. The enzyme from the cyanobacterium *Nodularia spumigena* NSOR10 (NsPPT) is the only one activating both cognate and noncognate CPs of cyanobacteria^26,27^. *Synechocystis* sp. PCC6803 (hereafter referred to as *Synechocystis*) encodes only one PPTase (SPPT) and this enzyme was shown to activate only the CP of its cognate FAS^27^. In addition, biochemical characterization of the PPTase from *Oscillatoria* PCC6506 (OPPT) was performed with one cognate CP^28^. On the other hand, the *E. coli* BAP1 strain carrying a chromosomal *Sfp* gene is able to produce several functional cyanobacterial FASs, PK synthases (PKSs) and NRPSs^29–31^, indirectly suggesting the versatility of Sfp in activating cyanobacterial CPs. Here, we report the characterization of six cyanobacterial PPTases and Sfp toward 11 CPs of known and silent pathways from cyanobacteria and *Streptomyces* strains. Biochemical and kinetic studies indicated the varying substrate promiscuity of these enzymes and suggested the coevolution of PPTases/cognate CP substrates. Two selected cyanobacterial PPTases and Sfp further demonstrated the versatile *in vivo* and *in vitro* catalytic activity when they were transiently expressed in *Synechocystis*. These results identify cyanobacterial PPTases with the catalytic proficiency and efficiency in activating CPs from diverse natural product biosynthetic pathways and lay a solid foundation to harness the biosynthetic potential of cyanobacteria via synthetic biology approaches.

## Materials and Methods

### Phylogenetic analysis of cyanobacterial PPTases

Sfp and *E. coli* AcpS were used as two query sequences to mine currently available cyanobacterial genomes in NCBI database (up to Nov 1st, 2016) using BLAST program. The output data of BLAST were carefully analyzed to identify the sequences with comparatively high similarity (with e-values ≤10^–5^) and to eliminate redundant sequences from taxonomically close species. The resulted selection of 39 PPTase protein sequences along with those from *Streptomyces rapamycinicus* NRRL5491, *Xenopus laevis* and *Homo sapiens* was aligned by Clustal Omega and then analyzed with MEGA7^32^ using a maxi-mum likelihood statistical method to construct a phylogenetic tree. The confidence was evaluated with 1000 bootstraps.

### Biochemical characterization of PPTase activity

Enzyme reaction solutions (100 μl) typically contained 50 mM Tris-Cl, pH 8.0, 12.5 mM MgCl_2_, 0.5 mM coenzyme A, 5 mM dithiothreitol (DTT) and 50 μM CPs. The reactions were initiated by adding 0.3 μM (final concentration) of PPTases, incubated at 37 °C for 20 min, and terminated by mixing with 100 μl of 10% formic acid. To quantitatively determine the relative activity of the enzymes, the reaction time can be up to 40 min. The quenched solutions were centrifuged at 4 °C, 16,000 × *g* for 15 min and clear supernatants were collected and subjected to HPLC and LCMS analysis with details shown in the supporting information. All experiments were repeated in triplicate. For kinetic studies, the reactions were set up as described above except that the concentrations of CPs were varied from 0.5 to 120 μM. The reactions were performed at 37 °C for 5-10 min to ensure that ≤10% of substrates were converted. To determine the concentrations of holo-CPs, 0.2 to 50 μM of apo-proteins were fully converted in the enzyme reactions and then quantitated in HPLC analysis to establish standard curves of holo-CPs. The concentrations of existing holo-CPs in the substrate solutions were subtracted in the data analysis. Data were fit into the Michaelis-Menten equation to determine kinetic parameters using GraphPad Prism 4.0. All experiments were independently repeated three times.

### Genetic engineering of *Synechocystis*

*Synechocystis* cells (about 1 × 10^8^ cells/ml) in the exponential phase were collected after centrifugation at 8,000 rpm for 15 min and resuspended in fresh BG11 medium at a density of 1 × 10^9^ cells/ml. Integration constructs at a final concentration of 10 μg/ml were then incubated with the cell solution at room temperature. After 5 h, the mixtures were spread onto BG11 agar plates supplemented with 5 μg/ml kanamycin. The segregation of wild type with the desirable mutants was achieved by iteratively streaking the colonies onto plates with progressively increased concentrations of kanamycin (up to 50 μg/ml). The final stable mutants were genotyped by the colony PCR using the primers listed in Table S1. Growth curves of the wild type and three mutant strains were determined by daily record of the OD_730_ of the liquid cultures over the period of 13 days.

### Quantitative RT-PCR analysis of the integrated exogenous PPTase genes

Total RNA samples were isolated from *Synechocystis* and its mutants using ZR Fungal/Bacterial RNA MiniPrep kit (Zymo Research). The quantity and quality of the isolated RNAs were determined using Nanodrop. Synthesis of cDNAs was performed with random primers following the manufacturer’s protocol (Thermo Scientific). The synthesized cDNAs were used as templates for qPCR to detect the transcription of the integrated PPTase genes, while the isolated RNA samples themselves were used as the templates of PCR reactions to detect any residual genomic DNAs using primers listed in Table S1. The student’s *t-test* analysis was applied to determine significance difference between the samples, and a *P*-value < 0.05 was considered to be statistically significant.

### Preparation of cell lysates of *Synechocystis* mutants

Cells of the wild type and three *Synechocystis* mutants were harvested from 0.8 to 1.0 L culture after centrifugation at 4 °C, 4,000 × *g* for 15 min. Cell pellets were washed with fresh BG11 medium and then resuspended in 4 ml of lysis buffer (50 mM MES, pH 7.0, 10 mM MgCl_2_, 5 mM CaCl_2_, 1 mM phenylmethylsulfonyl fluoride and 10% glycerol). The solutions were frozen at −80 °C and thawed at room temperature prior to the sonication on ice with 2-s pulses. Cell homogenates were centrifuged at 4 °C, 25,000 × *g* for 30 min to collect clear cell lysates. The enzyme reaction mixtures were set up as described above except containing 70 μl of cell lysates. The reactions were incubated at 37 °C for 16 h, and the holo-products were detected in LCMS analysis as described above. The reactions were performed in triplicate.

### Data availability statement

All data generated or analyzed during this study are included in this manuscript (and its Supplementary Information files).

## Results and Discussion

### Phylogenetic analysis of cyanobacterial Sfp-type PPTases

**Figure 1:**
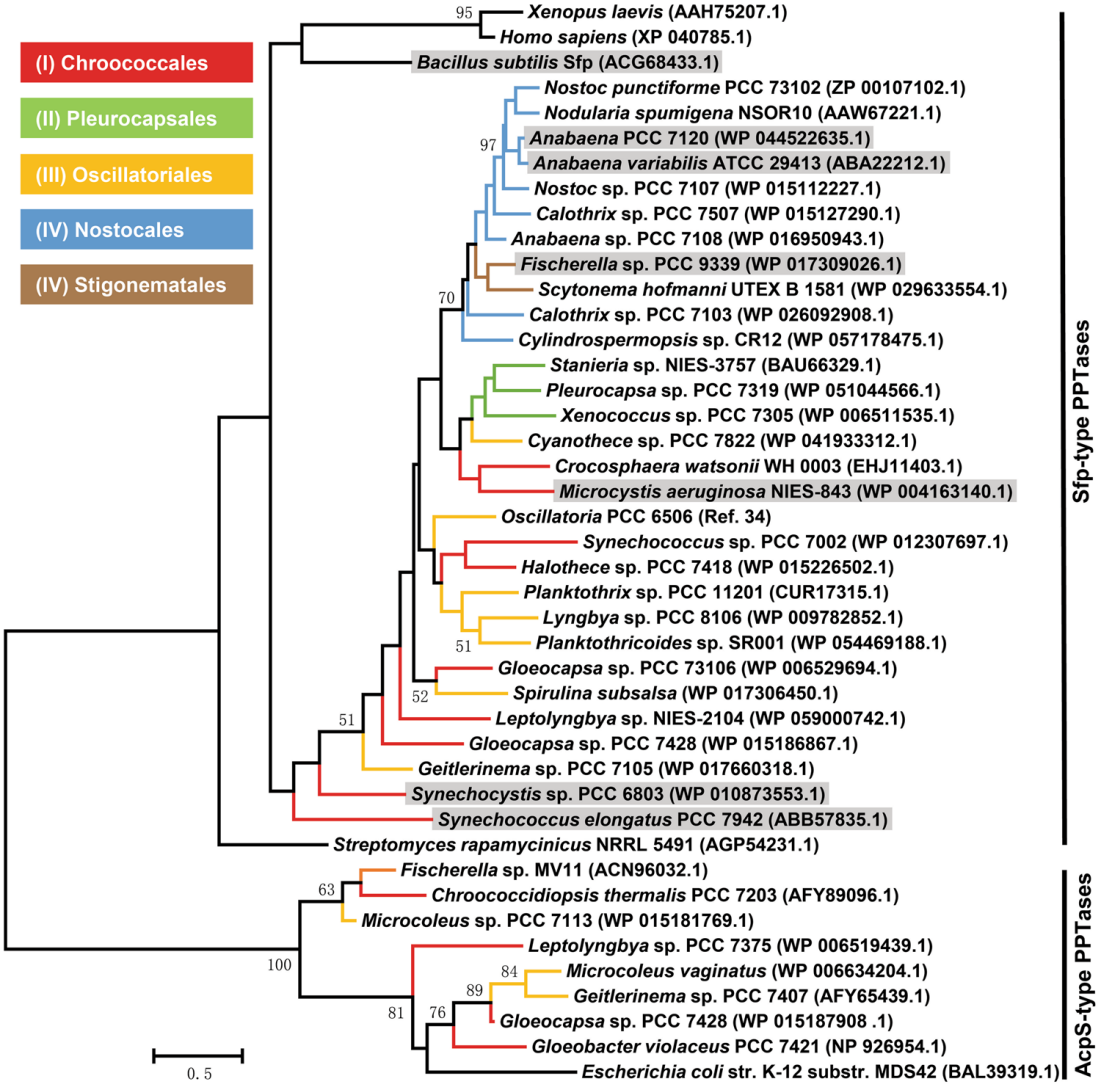
A maximum-likelihood phylogeny based on selected cyanobacterial PPTases. A phylogenetic tree was generated using MEGA7 maximum-likelihood method. Enzymes were shown as the names of corresponding strains with NCBI accession numbers given in parenthesis. Branches were color-coded according to morphological subsections of cyanobacteria. Branch length is proportional to the amount of genetic change. Significant bootstrap values (over 500 of 1,000 repeats) are shown. PPTases with shaded taxa names were selected for the characterization in this study.

To gain an understanding of the evolutionary relationship of cyanobacterial PPTases, we mined all cyanobacterial genomes available in NCBI database using Sfp and *E. coli* AcpS as queries. A total of 39 sequences was then selected and retrieved from 39 strains of 26 genera that broadly cover all five subsections of cyanobacteria (Table S2)^33^. Phylogenetic analysis of these sequences led to a constructed tree comprising an AcpS-type clade, which included AcpS and eight cyanobacterial PPTases, and a Sfp-type clade containing all other enzymes (Fig. 1)^19^. In the Sfp-type clade, Sfp and three PPTases from *Streptomyces rapamycinicus* NRRL5491, *Xenopus laevis* and *Homo sapiens* as outgroups were separated from cyanobacterial Sfp-type PPTases. Furthermore, enzymes from the heterocystous cyanobacteria (subsections IV and V) formed a sub-clade, while those from sections I-III showed a less clear pattern. For example, the PPTases from *Gloeocapsa* sp. PCC73106 (subsection I) and *Spirulina subsalsa* (subsection III) fell into the same group (Fig. 1). These results indicate that cyanobacterial Sfp-type PPTases share a common ancestor and have acquired different traits over the course of evolution.

### Selection of cyanobacterial Sfp-type PPTases and CP substrates

To comprehensively understand the catalytic performance of cyanobacterial Sfp-type PPTases, we next selected representative enzymes based on the result of phylogenetic analysis (Fig. 1) and predicted biosynthetic potential of cyanobacterial strains. The genomes of *Fischerella* sp. PCC9339 (hereafter *Fischerella*), *Anabaena* sp. PCC7120 (hereafter *Anabaena*) and *A. variabilis* ATCC29413 all possess more than 10 NRP and/or PK gene clusters^13^ and their PPTases, FPPT, APPT and AvPPT, respectively, were included in this work due to potentially broad substrate promiscuity. In addition, FPPT and APPT/AvPPT belong to two distantly related groups in the same subclade of subsections IV and V, likely representing the enzymes from a variety of heterocystous cyanobacteria (Fig. 1). We also selected the PPTases from *Microcystis aeruginosa* NIES843 (MPPT) carrying a rich biosynthetic potential^34^ and *Synechococcus elongatus* PCC7942 (SePPT) that forms a separate leaf in the phylogenetic tree (Fig. 1). *S. elongatus* PCC7942 encodes no PK or NRP cluster. Furthermore, SPPT was included as a control due to its reported narrow substrate scope^27^. Finally, the paucity of biochemical characterization of Sfp in activating cyanobacterial CPs led to its selection. The six selected cyanobacterial PPTases and Sfp contain the featured W/KEA motif^22^ (Fig. S2) and together cover the broad space of the constructed phylogenetic tree (Fig. 1).

We next chose 11 CPs from multiple biosynthetic pathways of different species for biochemical characterization of the selected PPTases (Table S3, Fig. S3). They included two ACPs of FASs from *Synechocystis* (SFACP) and *Anabaena* (AFACP), one ACP of the glycolipid PKS in *Anabaena* (APACP)^35^, one ACP of the apratoxin (PK/NRP) gene cluster in *Lyngbya (Moorea) bouillonii* (AprACP), and the PCP of the shinorine gene cluster from *Fischerella* ^36^. In addition, we included three CPs of uncharacterized gene clusters from *Fischerella* (FNPCP, an NRP pathway), *Anabaena* (APNPCP, an NRP/PK pathway) and *M. aeruginosa* NIES843 (MACP, an NRP/PK pathway) and one ACP from *Fischerella* (FNsACP) that is a homologous enzyme of previously characterized ArCP_Np_^26^. To thoroughly examine the versatility of selected PPTases, we also included one ACP of a putative concanamycin gene cluster from *Streptomyces coelicolor* A(3)2 (ScACP) and one PCP of a thaxtomin cluster from the plant pathogen *S. scabiei* 87.22 (SsPCP)^37^.

### *In vitro* Phosphopantetheinylation of cognate and noncognate CPs by selected PPTases

**Figure 2:**
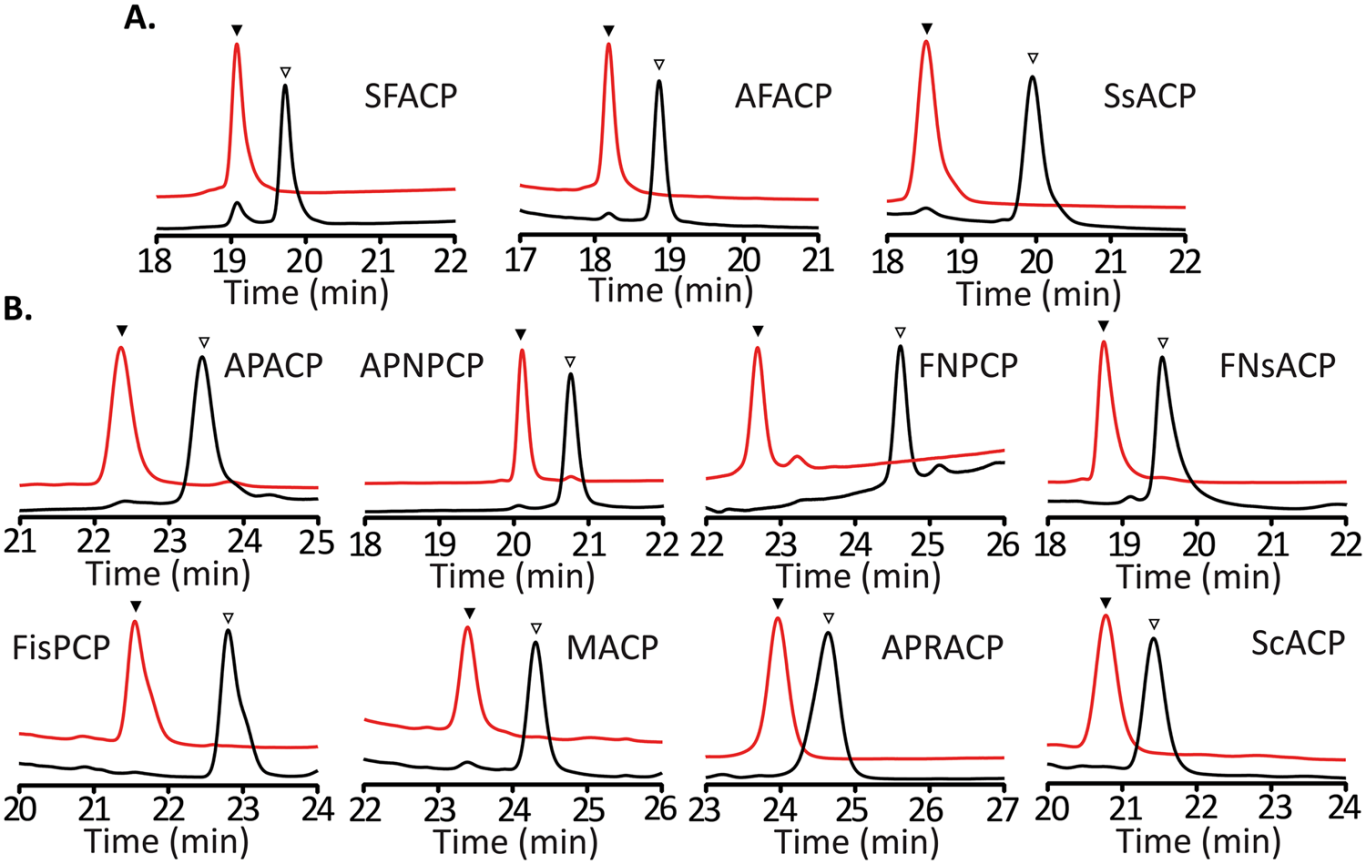
HPLC traces of APPT reactions with 11 CP substrates. Red traces represented the enzyme reactions and black ones showed the substrates. ▼ indicated the holo-CP, while ∇ represented the apo-substrate. (**A**) SFACP, AFACP and SsPCP were partially phosphopantetheinylated over the course of overexpression in *E. coli*, and were fully converted in the enzyme reactions. (**B**) All other CPs were in apo-forms and completely functionalized in the APPT reactions. Similar traces were observed in the reactions of several other PPTases.

Recombinant proteins (Fig. S3) were purified from *E. coli* by a single step Ni-NTA affinity chromatography. All purified proteins showed expected molecular weights in SDS-PAGE analysis (Fig. S4), and the CP substrates were further validated in LC-MS analysis. SFACP, AFACP and SsPCP gave rise to two peaks in the LC traces with the minor peaks as the holo-proteins (Fig. 2A). The rest of CP substrates were in the apo-form (Fig. 2B, Table S3, and Fig. S5). This result suggests that *E. coli* AcpS activates noncognate ACPs of FASs to a low level and shows the limited promiscuity toward CPs of NPRSs and PKSs.

**Figure 3:**
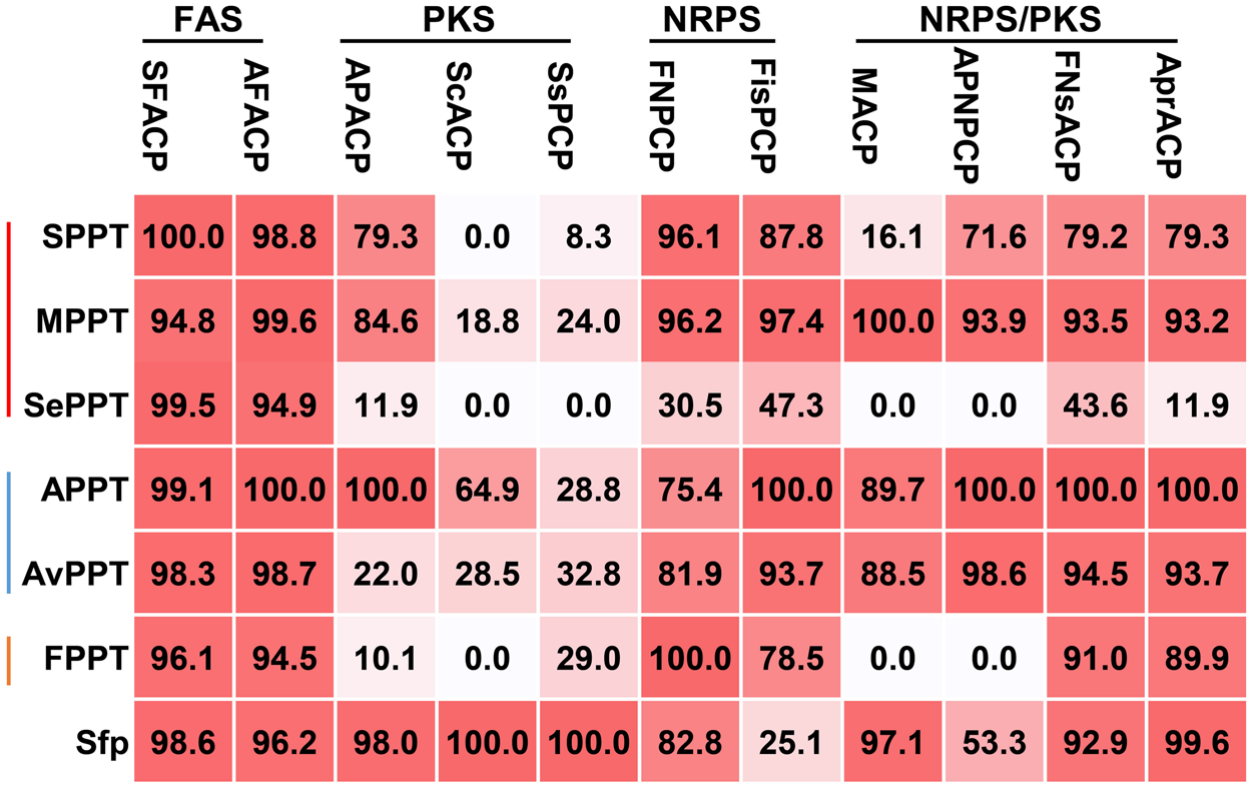
Relative activities of seven PPTases toward 11 CP substrates. The activity of the most active PPTase of a CP was set as 100%, and those of other PPTases toward the same substrate were normalized as shown in the heatmap. The data represented the mean of three independent experiments. Red to white color indicated the high to low relative activities of PPTases. CPs were grouped according to the biosynthetic pathways while cyanobacterial PPTases were organized by the subsections of sources.

We next examined the catalytic activity of each PPTase toward all 11 recombinant CPs. The substrates in 69 out of all 77 reactions were fully converted into the holo-products (Figs 2 and 3). APPT, AvPPT, MPPT and Sfp functionalized all substrates (Table S4). Unexpectedly, SPPT also phosphopantetheinylated all CP substrates except ScACP (Fig. 3, and Table S3). In an early report, SPPT was unable to activate two ACPs from *Nostoc punctiforme* ATCC 29133 and one PCP from *M. aeruginosa* PCC7806^27^. On the other hand, SePPT and FPPT showed no activity toward APNPCP, MACP and ScACP and the former was also inactive toward SsPCP.

To quantitate the substrate preference of PPTases, the reactions were performed to allow less than 95% of substrate conversion. After setting the activity of the most active enzyme as 100%, the activities of other PPTases toward the same substrate were normalized (Fig. 3, and Table S4). By this analysis, APPT showed the highest conversion rates toward seven cyanobacterial CPs from the FA, PK, NRP, and PK/NRP biosynthetic pathways. APPT also activated ScACP and SsPCP from *Streptomyces* species with relative activities of 64.9% and 28.8%, respectively, demonstrating a broad-range substrate scope. In line with the observed substrate flexibility, APPT has functionalized the ACP of microalgal polyunsaturated fatty acid synthase in canola^38^ and allowed the hetero-logous production of lyngbyatoxin A in *Anabaena* ^17^. AvPPT possessed a similar substrate scope and comparable activities to APPT. We also observed the high activity and versatility of MPPT and SPPT. MPPT showed more than 84% relative activities toward all nine cyanobacterial CPs and a modest relative activity toward ScACP (18.8%) and SsPCP (24%), while SPPT promoted more than 71% relative conversions of eight cyanobacterial CPs. Similar to the above four cyanobacterial PPTases, Sfp functionalized seven cyanobacterial CPs (25.1% to 99.6%) to comparable levels of APPT and showed the highest relative activities toward ScACP and SsPCP. By contrast, SePPT and FPPT in general showed lower relative activities in functionalizing 7 and 8 selected CPs, respectively. Collectively, the results of biochemical studies provide the first comprehensive evaluation of cyanobacterial PPTases in terms of enzymatic activity and substrate scope.

### Kinetic analysis of APPT, MPPT, SPPT and Sfp

To assess the catalytic efficiency of selected PPTases, we kinetically analyzed APPT, MPPT, SPPT and Sfp in activating all 11 CP substrates (Table 1, and Fig. S6). The conversion of SsPCP by Sfp showed the highest catalytic efficiency (*k*_cat_/*K*_m_ = 2.1 ± 0.2 µM^−1^ min^−1^) of all reactions, consistent with the overall kinetic performance of Sfp toward CPs of actinomycetes^21,39^. On the other hand, the catalytic efficiencies (*k*_cat_/*K*_m_) of Sfp toward nine cyanobacterial CPs were varied from 0.1 ± 0.02 µM^−1^ min^−1^ (FisPCP) to 1.5 ± 0.3 µM^−1^ min^−1^ (APACP).

**Table 1:** Kinetics parameters of four selected PPTases toward 11 CPs^a^.

Substrate	APPT			MPPT			SPPT			Sfp		
	*K*_m_^b^	*k*_cat_^b^	*k*_cat_/*K*_m_^b^	*K*_m_	*k*_cat_	*k*_cat_/*K*_m_	*K*_m_	*k*_cat_	*k*_cat_/*K*_m_	*K*_m_	*k*_cat_	*k*_cat_/*K*_m_
SFACP	2.8 ± 0.2	1.6 ± 0.09	0.6 ± 0.07	3.2 ± 0.2	1.2 ± 0.1	0.4 ± 0.07	1.5 ± 0.2	1.4 ± 0.1	0.9 ± 0.2	2.5 ± 0.3	2.2 ± 0.1	0.9 ± 0.2
AFACP	6.8 ± 0.5	11.7 ± 0.5	1.7 ± 0.2	6.9 ± 0.4	8.4 ± 0.2	1.2 ± 0.1	13.3 ± 1.6	14.7 ± 1.1	1.1 ± 0.2	11.6 ± 0.6	9.8 ± 0.3	0.8 ± 0.07
APACP	10.0 ± 0.9	17.0 ± 0.6	1.6 ± 0.2	23.1 ± 4.1	21.2 ± 1.6	0.9 ± 0.2	26.5 ± 5.2	14.6 ± 1.3	0.5 ± 0.1	15.3 ± 2.1	22.1 ± 1.4	1.5 ± 0.3
ScACP	14.1 ± 1.6	5.4 ± 0.3	0.4 ± 0.07	12.4 ± 1.5	2.6 ± 0.1	0.2 ± 0.04	N/A^c^	N/A^c^	N/A^c^	8.3 ± 0.5	15.2 ± 0.5	1.8 ± 0.1
SsPCP	7.4 ± 0.5	7.6 ± 0.2	1.0 ± 0.09	9.1 ± 0.3	7.1 ± 0.1	0.7 ± 0.04	14.3 ± 1.1	1.0 ± 0.04	0.06 ± 0.008	7.9 ± 0.7	16.7 ± 0.7	2.1 ± 0.2
FNPCP	12.1 ± 0.5	12.2 ± 0.3	1.0 ± 0.07	7.2 ± 0.7	9.6 ± 0.4	1.3 ± 0.2	7.0 ± 0.6	10.1 ± 0.4	1.4 ± 0.2	11.7 ± 0.8	14.5 ± 0.6	1.2 ± 0.1
FisPCP	7.1 ± 0.5	2.3 ± 0.06	0.3 ± 0.03	7.2 ± 0.3	1.9 ± 0.03	0.3 ± 0.02	10.0 ± 0.5	2.5 ± 0.05	0.3 ± 0.02	14.0 ± 1.2	1.7 ± 0.07	0.1 ± 0.02
MACP	7.9 ± 0.9	3.7 ± 0.2	0.5 ± 0.08	4.9 ± 0.3	4.2 ± 0.1	0.9 ± 0.07	22.0 ± 1.6	4.3 ± 1.1	0.2 ± 0.06	6.7 ± 0.7	5.1 ± 0.2	0.8 ± 0.1
APNPCP	1.6 ± 0.1	1.0 ± 0.05	0.6 ± 0.09	9.5 ± 0.4	1.9 ± 0.1	0.2 ± 0.02	17.0 ± 1.3	2.3 ± 0.2	0.1 ± 0.02	12.0 ± 0.9	2.2 ± 0.2	0.2 ± 0.02
FNsACP	8.4 ± 0.8	2.2 ± 0.08	0.3 ± 0.03	8.3 ± 0.8	2.1 ± 0.4	0.3 ± 0.07	14.1 ± 1.4	1.5 ± 0.07	0.1 ± 0.02	10.9 ± 0.5	2.4 ± 0.05	0.2 ± 0.01
AprACP	7.8 ± 0.8	14.3 ± 0.8	1.8 ± 0.3	9.1 ± 1.4	10.4 ± 0.8	1.1 ± 0.3	12.9 ± 2.1	13.1 ± 1.2	1.0 ± 0.2	9.0 ± 1.0	2.9 ± 0.1	0.3 ± 0.05

^a^The data represented mean ± SD of three independent experiments; ^b^Units of *K*_m_, *k*_cat_, and *k*_cat_/*K*_m_ are µM, min^−1^, and µM^−1^ min^−1^, respectively; ^c^No detectable activity.

Among the selected PPTases, APPT demonstrated the highest catalytic efficiencies toward AprACP, AFACP and APACP (1. 6 to 1.8 µM^−1^ min^−1^) (Table 1). These three cyanobacterial substrates were also favored by MPPT and SPPT (*k*_cat_/*K*_m_ ≥ 1.0 µM^−1^ min^−1^). The catalytic efficiencies of APPT, MPPT and SPPT toward other cyanobacterial substrates were varied but both FisPCP and FNsACP were clearly disfavored (*k*_cat_/*K*_m_ = 0.1 to 0.3 µM^−1^ min^−1^). Of note, we discovered that cognate CP/PPTase pairs generally demonstrate high catalytic efficiencies (*k*_cat_/*K*_m_ ≥ 0.9 µM^−1^ min^−1^, e.g., MACP/MPPT), likely indicating the potential co-evolution of biosynthetic enzymes.

The *K*_m_ values of four PPTases toward 11 CPs were in the µM range (Table 1). SFACP showed relatively tight interactions with all PPTases (*K*_m_ = 1.5 to 3.2 µM), while APACP was a relatively weak binder (*K*_m_ = 10.0 to 26.5 µM). Conversely, the PPTases showed the higher activity toward APACP (*k*_cat_ ≥ 14.6 ± 1.3 min^−1^) compared to SFACP (*k*_cat_ ≤ 2.2 ± 0.1 min^−1^). Interestingly, CP substrates demonstrated the lowest *K*_m_ values toward their cognate PPTases in comparison with other enzymes, indicating the potential co-evolution. In this regard, SPPT showed higher *K*_m_ values toward the majority of noncognate CPs than APPT and MPPT, presumably because of the lack of any PK or NRP cluster in *Synechocystis*. In line with this observation, the interactions of Sfp with the majority of cyanobacterial CPs were also relatively weak.

### *In vivo* and *in vitro* activity of transiently expressed APPT, MPPT and Sfp in *Synechocystis*

**Figure 4:**
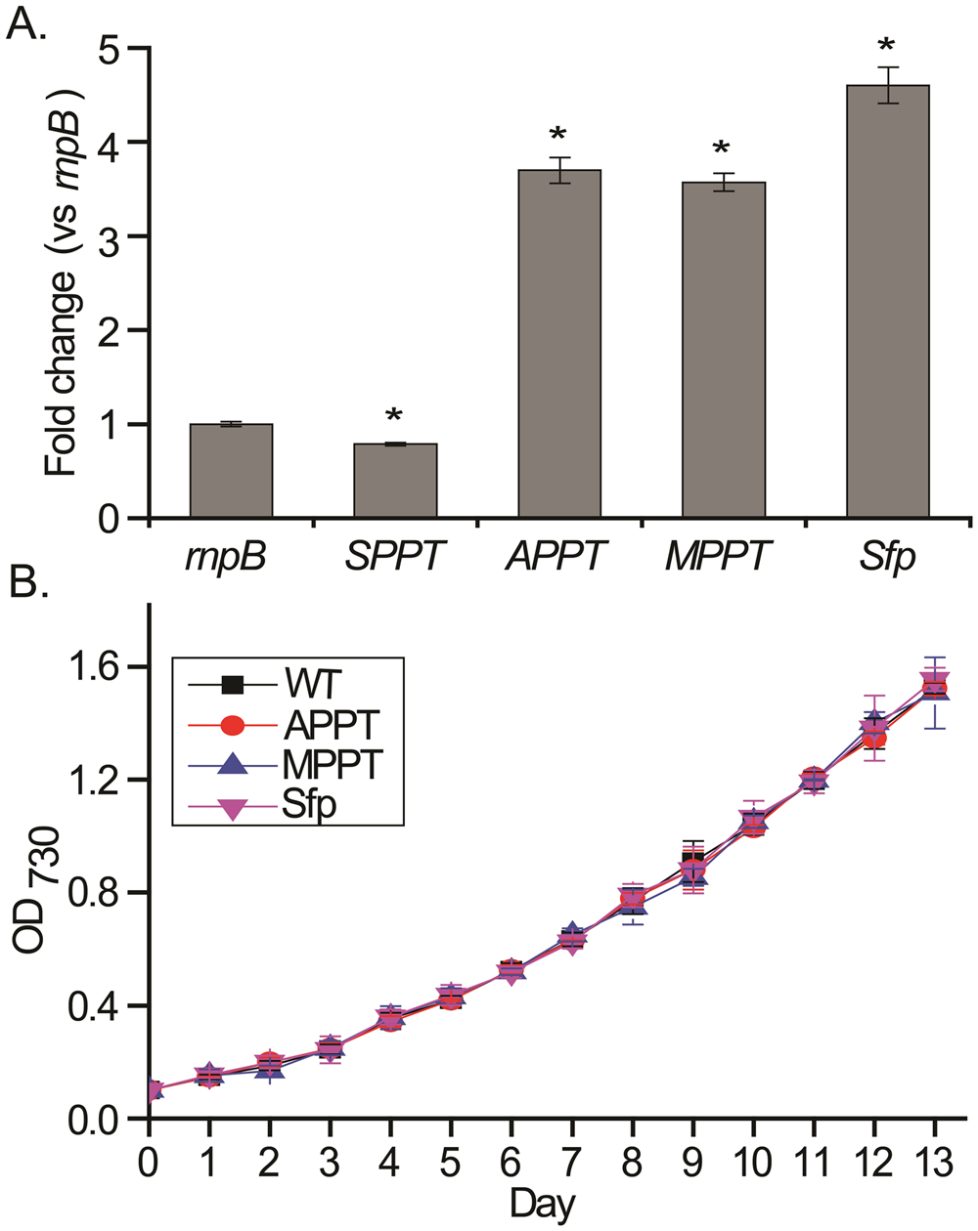
The *in vivo* activity of APPT, MPPT and Sfp in *Synechocystis* mutants. (**A**) Quantitative analysis of transcriptional levels of *SPPT*, *APPT*, *MPPT* and *Sfp* genes. The signals were normalized with that of *rnpB* gene from each mutant and wild type. The asterisk (*) indicated statistical significance differences (P < 0.05). The error bars represented the standard deviations of triplicate assays. (**B**) Growth curve of *Synechocystis* wild type and mutants. OD_730_ was continuously monitored for 13 days. The error bars represented the standard deviations of triplicate measurements.

**Figure 5:**
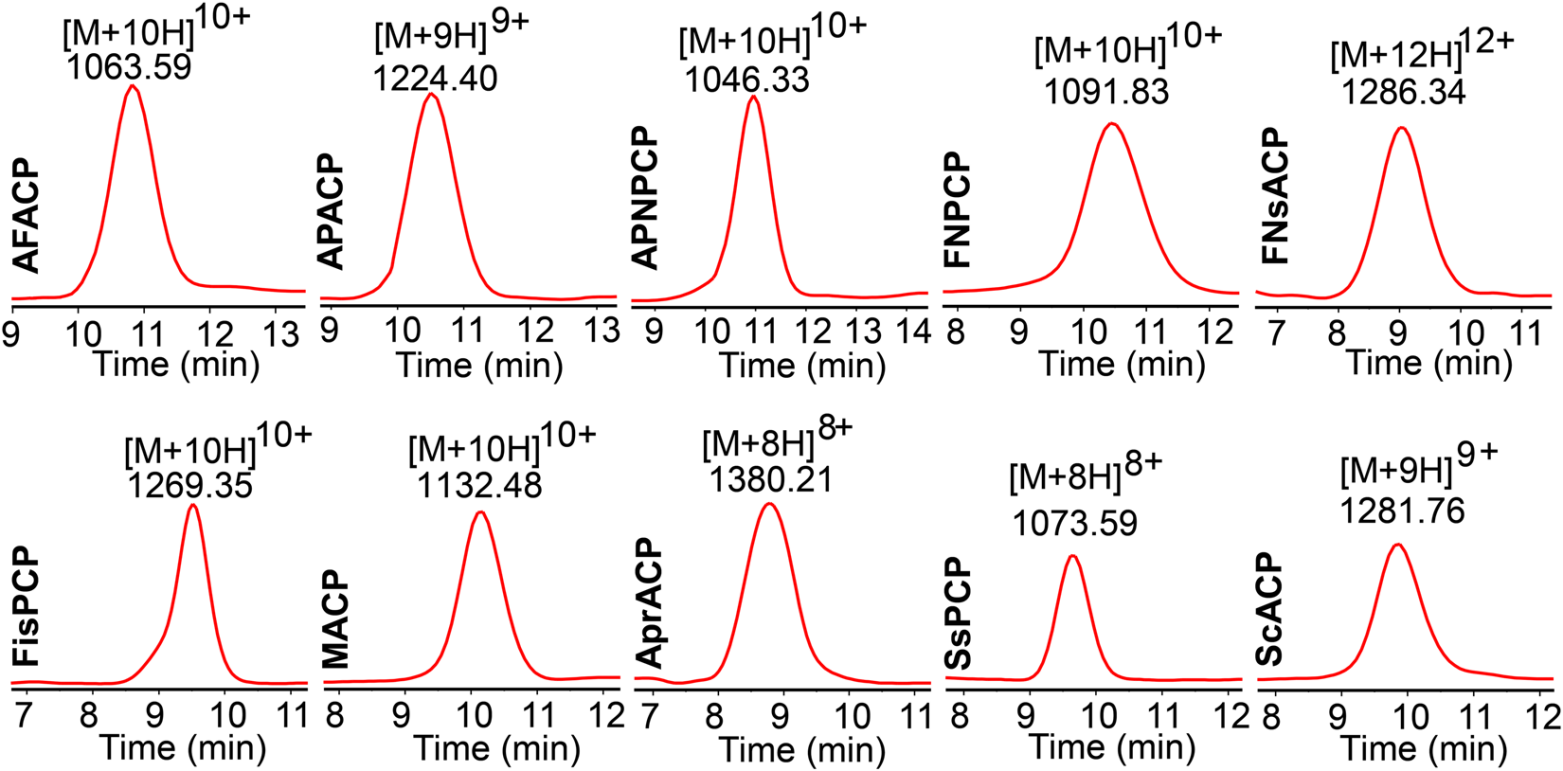
Extracted ion chromatograms of holo-products in the reactions of cell lysate of *Synechocystis APPT* mutant. The products showed the expected molecular weights. Similar traces were observed in the reactions using cell lysates of two other mutants.

To explore the *in vivo* catalytic performance of APPT, MPPT and Sfp, we chromosomally replaced the *SPPT* gene, the only known *PPTase* gene in *Synechocystis*, with their genes (Figs S7 and S8). The expression of the integrated PPTase genes was controlled by a constitutive strong promoter Ptrc^40^. After homologous recombination and multiple rounds of segregation, three stable *Synechocystis* mutants were identified by the PCR diagnosis as the loss of the *SPPT* gene and the presence of foreign *PPTase* gene (Fig. S8A). The transcription of these *PPTase* genes was confirmed in RT-PCR analysis (Fig. S8B). Furthermore, their transcription levels were quantitated in qRT-PCR analysis after being normalized with that of the *rnpB* gene encoding the RNA subunit of RNase P in each engineered strain and wild type. This analysis found that the transcriptional levels of the foreign *PPTase* genes in the mutants are five to six times higher than that of *SPPT* in the wild type (Fig. 4A). Importantly, the growth curve of the three mutant strains closely resembled the wild type over the entire 13-day culturing period (Fig. 4B). This data indicates the *in vivo* function of APPT, MPPT and Sfp as activating SFACP for the synthesis of essential FAs. To further evaluate the catalytic performance of these enzymes transiently expressed in cyanobacteria, we employed the soluble cell lysates of three *Synechocystis* mutants to functionalize all CP substrates except SFACP. LC-MS analysis detected the holo-products from all 30 reactions that were incubated for 16 hours (Fig. 5). The relatively long reaction time is likely caused by the low concentrations of recombinant proteins from the cyanobacterial expression system^41^. A high-copy self-replicating vector can potentially alleviate this issue^42^. Similar to an early report^27^, our attempts to create a *Synechocystis* Δ*SPPT* mutant failed, which further indicates the indispensability of a functional PPTase to fatty acid synthesis. Nonetheless, these *in vitro* results confirmed the catalytic functions of APPT, MPPT and Sfp expressed in *Synechocystis* mutants. The three *Synechocystis* mutants can be useful for the heterologous production of various cyanobacterial PKs, NRPs, and their hybrids.

## Conclusions

PPTases are essential enzymes of all three domains of life as they functionalize CPs of FASs, PKSs, and NRPSs. The past two decades have witnessed significant advances of PPTase research, particularly about structure-function-relationship and biotechnological and biomedical applications^19^. Here, we characterized the phylogenetic relationships of cyanobacterial PPTases and then rationally selected six Sfp-like cyanobacterial enzymes along with Sfp to characterize their substrate scope and catalytic activity toward 11 CPs of FASs, PKSs, and NRPSs from cyanobacteria and *Streptomyces* strains. APPT, MPPT and Sfp demonstrated high catalytic activity and kinetic performance toward the majority of cyanobacterial CPs. Interestingly, PPTases favored cognate CP substrates in their reactions, suggesting the use of proper (e.g., cognate) PPTase for the heterologous production of natural products. This work used standalone CP substrates to characterize the catalytic performance of PPTases and previous studies suggest that they can demonstrate similar activity profiles toward CP domains within intact NRPS, PKS or their hybrids^43,44^. Furthermore, the validated *in vivo* and *in vitro* functions of transiently expressed APPT, MPPT and Sfp in the *Synechocystis* mutants suggest the availability of the novel, capable cyanobacterial synthetic biology chassis for the production of primary and secondary metabolites of cyanobacteria.

## Electronic supplementary material

Supporting materials
