# Supplementary material for: Cyanobacterial Sfp-type phosphopantetheinyl transferases functionalize carrier proteins of diverse biosynthetic pathways
Source: Sci Rep. 2017 Sep 19;7:11888. doi: 10.1038/s41598-017-12244-3 (PMC5605751; doi:10.1038/s41598-017-12244-3)
Supplement: Supplementary file 1 — Supporting materials [file 41598_2017_12244_MOESM1_ESM.pdf]

Supplementary information:

Cyanobacterial Sfp-type phosphopantetheinyl transferases functionalize carrier proteins of diverse biosynthetic pathways

Guang Yang,[†] Yi Zhang,[†] Nicholas K. Lee,[†] Monica A. Cozad,[†] Sara E. Kearney,[†,§] Hendrik Luesch,[†] and Yousong Ding[†,*]

[†]Department of Medicinal Chemistry and Center for Natural Products, Drug Discovery and Development, University of Florida, Gainesville, FL 32610, USA
[§]NIH Chemical Genomics Center, National Center for Advancing Translational Sciences, National Institutes of Health, Rockville, MD 20850, USA

Table of Contents

1. Methods.
2. Table S1: Oligos used in the present study.
3. Table S2: PPTases used in the phylogenetic analysis.
4. Table S3: Selected CPs for the characterization of PPTases.
5. Table S4: Relative activity of seven PPTases in activating 11 CPs.
6. Fig. S1: Schematic representation of post-translational phosphopantetheinylation of a CP domain by a PPTase.
7. Fig. S2: Multiple-sequence alignment of characterized cyanobacterial PPTases and Sfp.
8. Fig. S3: Sequences of selected *CP* genes and codon optimized *Sfp* gene.
9. Fig. S4: SDS-PAGE analysis of purified PPTases and CP proteins.
10. Fig. S5: HR-MS spectra of apo- and holo-CPs that were used and produced in the PPTase reactions.
11. Fig. S6: Michaelis-Menten kinetic analysis of APPT evaluated for 11 selected CP substrates.
12. Fig. S7: Schematic representation of homologous replacement of the *SPPT* gene with foreign *PPTase* genes in *Synechocystis* sp. PCC6803.
13. Fig. S8. PCR analyses of the integration and transcription of the *APPT*, *MPPT* and *Sfp* genes in *Synechocystis* mutants.

Methods:

Reagents and chemicals

Restriction enzymes, *Taq* DNA polymerase and Phusion DNA polymerase were purchased from Thermo Scientific. The protein ladders were purchased from Thermo Scientific or NEB. Chemicals and solvents were from Sigma Aldrich, Fisher Scientific or RPI Corp (USA). The GeneJET Plasmid Miniprep Kit, PCR Purification Kit and Gel Extraction Kit were from Thermo Scientific. Oligonucleotide primers were synthesized by Sigma Aldrich, while codon-optimized *Sfp* gene was obtained from GenScript. DNA sequencing was performed at Eurofins.

Strains and culture conditions

Escherichia coli DH5α and BL21-CodonPlus (DE3) RIPL were used for routine molecular biology studies and protein expression, respectively, and were grown in Luria-Bertani broth or Terrific broth. *Synechocystis* sp. PCC6803, *Anabaena* sp. PCC7120, *Anabaena variabilis* ATCC29413, *Fischerella* sp. PCC9339, *Microcystis aeruginosa* NIES-843, and *Synechococcus* sp. PCC7942 were purchased from UTEX or NIES (Japan) and cultured in BG11 medium with CO_2 bubbling. All cyanobacterial cultures were performed at 26 °C with 16h/8h light/dark cycle using 2000-2500 lux during lighting period. BG-11 medium supplemented with 1.0% (wt/vol) agar and 0.3% (wt/vol) sodium thiosulfate was used to grow cyanobacterial strains on the plate.

Construction of plasmids

All oligonucleotide primers used in this work were listed in **Table S1**. The *PPTase* and *CP* genes were PCR amplified and cloned into pET28b (Novagen) to generate the expression plasmids. The inserts in the integration plasmids were sequentially constructed in the PCR reactions as shown in **Fig. S6**. The details were included in the supporting information. Briefly, a T7 terminator fragment was first introduced to the 3'-end of *APPT*, *MPPT* and *Sfp* genes by the PCR reactions. The resulted amplicons were fused to the 3'-end of Ptrc promoter-ribosomal binding site (Ptrc-RBS) in the overlapping PCR reaction. Next, a kanamycin resistance cassette amplified from pUC4K (Pharmacia) was fused to the 3'-end of the above amplicons to generate the final insert products. The final products were cloned into the integration vector pUC19int. To generate the pUC19int, the upstream and downstream regions (~1 kb) of the *SPPT* gene in *Synechocystis* were amplified and fused in the PCR reactions. The fusion product was then digested by *Hind*III and *Eco*RI and cloned into pUC19 to create pUC19int. All constructed plasmids were sequenced to eliminate potential errors in the inserts.

Protein expression and purification

Recombinant proteins with an N-terminal His-tag were expressed in *E. coli* BL21-CodonPlus (DE3) RIPL. Cells were grown at 37 °C to an OD600 = 0.5-0.6, and then cooled to 18 °C prior to the addition of 0.1-0.5 mM isopropyl-β-D-galactopyranoside (IPTG). The cultures were grown at 18 °C for another 18-20 h before harvesting. *E. coli* cells were collected after centrifugation at 4 °C, 4,000 x g for 15 min, and frozen at -80 °C until the use. Pellets were thawed on ice, resuspended in a suitable volume of lysis buffer (50 mM Tris-HCl buffer, pH 8.0, 300 mM NaCl, 3 mM BME, 10 mM imidazole, 10% glycerol; wt/vol = 1:4), and subjected to sonication on ice with 2-s pulses. The soluble fractions were collected after centrifugation at 4 °C, 25,000 x g for 30 min, and incubated with Ni-NTA agarose resin (Thermo Fisher) at 4 °C for 1 h. The resin was then washed successively with ~10 column volumes of the lysis buffer containing 30 mM imidazole. Recombinant proteins were eluted with 50-300 mM imidazole in the lysis buffer. Proteins were analyzed with 15% SDS-PAGEs that were stained with Coomassie blue for imaging. After SDS-PAGE analysis, elution fractions containing the targeted proteins were combined. The purified

proteins were then exchanged into a storage buffer (50 mM Tris-HCl buffer, pH 8, 100 mM NaCl, 10% glycerol) using PD-10 column according to the manufacture's protocol (GE), aliquoted and stored at -80 °C until the use. The concentrations of recombinant proteins were determined by Nanodrop and/or Bradford assay.

HPLC and LC-MS analysis

A Shimadzu Prominence UHPLC system (Kyoto, Japan) fitted with a Vydac 218TP54-C18 (5 µm, 4.6 mm x 250 mm) column was used for HPLC analysis. Solvent A was H_2O with 0.1% TFA and solvent B was CH_3CN with 0.1% TFA. The column was equilibrated with 10% solvent B for 2 min and then protein sample was eluted with a linear gradient of 10-70% in 30 min, followed by another linear gradient of 70-98% solvent B in 1 min. The column was further cleaned with 98% solvent B for 5 min and then re-equilibrated with 10% solvent B for 2 min. The flow rate was set as 0.8 mL/min, and the product was detected at 220 nm with a PDA detector. Apo- and holo-proteins were further analyzed in LC-MS analysis. MS spectra were acquired by using an API Qstar Pulsar i hybrid tandem mass spectrometer (Applied Biosystems). An Agilent Eclipse Plus C18, (3.5 um, 2.1 x 100 mm) was used. In LC-MS analysis, solvent A was H_2O with 0.1% FA and solvent B was CH_3CN with 0.1% FA. The protein samples were eluted with a linear gradient of 10-90% in 15 min at a flow rate of 0.3 mL/min. HRMS data were obtained using a Thermo Fisher Q Exactive Focus mass spectrometer equipped with electrospray probe on Universal Ion Max API source. The LC conditions were the same as those for the LC-MS analysis.

Table S1. Oligos used in the present study.

Primers	Sequence 5'-3'	Function
Sfp-Fw	CATGCCATGGAAATTTATGGGATTTAC	Sfp expression
Sfp-Rv	CCGCTCGAGCTACAACAGTTCTTCATAG	Sfp expression
MPPT-Fw	CATGCCATGGTTATATCTACCGATGA	MPPT expression
MPPT-Rv	CCGCTCGAGTAGATCAGAAAGGCCA	MPPT expression
SPPT-Fw	CATGCCATGGTCCCCCAGCCCCAAAT	SPPT expression
SPPT-Rv	CCGCTCGAGGGGCAATGAATCAAGG	SPPT expression
SePPT-Fw	CATGCCATGGAACGCCCCAACCCTAG	SePPT expression
SePPT-Rv	CCGCTCGAGATGATTTTTCCGGATTATG	SePPT expression
APPT-Fw	CATGCCATGGTGCAGCATACTTGGC	APPT expression
APPT-Rv	CCGCTCGAGATAATGCCAGAATTTTG	APPT expression
AvPPT-Fw	CATGCCATGGTGCAGCATACTTGGCTAC	AvPPT expression
AvPPT-Rv	CCGCTCGAGATACTGCCAGAATTTTGGC	AvPPT expression
FPPT-Fw	CATGCCATGGGGTCTGAGACTAATCA	FPPT expression
FPPT-Rv	CCGCTCGAGATACTGCCAGTACTTTAA	FPPT expression
SFACP-Fw	CATGCCATGGATCAGGAAATTTTTGA	SFACP expression
SFACP-Rv	CCGCTCGAGTTTACTTTCGATATGCTC	SFACP expression
AFACP-Fw	GGAATTCCATATGAGCCAATCAG	AFACP expression
AFACP-Rv	CCGCTCGAGAGCTGATGCGGCAACTTG	AFACP expression
APACP-Fw	CATGCCATGGGTCTAAAACAAAATTATAG	APACP expression
APACP-Rv	CCGCTCGAGAGATTGTTCTTCCAATTCTTC	SFACP expression
APNPCP-Fw	CATGCCATGGAACAATCTACAACTAATC	APNPCP expression
APNPCP-Rv	CCGCTCGAGATCAGTAATAGGCGATTG	APNPCP expression
FNPCP-Fw	CATGCCATGGCCCAACGCCCTATCATTATC	FNPCP expression
FNPCP-Rv	CCGCTCGAGTTCAACTTCATCACTATC	FNPCP expression
FisPCP-Fw	CATGCCATGGGATCGCTTCCCAAACCTG	FisPCP expression
FisPCP-Rv	CCGCTCGAGTGAATTGGGAAAAACATC	FisPCP expression
FNsACP-Fw	CATGCCATGGCTTTTCTAGAAGATGTC	FNsACP expression
FNsACP-Rv	CCGCTCGAGGGAATTACCTAGAAAAGC	FNsACP expression
AprACP-Fw	CATGCCATGGAAATTTTTGAACAGGAAT	AprACP expression
AprACP-Rv	CCGCTCGAGACTAAAATTAATATCTTC	AprACP expression
MACP-Fw	CATGCCATGGTGACAACTGTTCAATC	MACP expression
MACP-Rv	CCGCTCGAGAAGATATAATTCCCCT	MACP expression
ScACP-Fw	CATGCCATGGAGCAGCGGCTGGCTC	ScACP expression
ScACP-Rv	CCGCTCGAGCTCCTGCTCGCCGAAC	ScACP expression
SSPCP-Fw	CATGCCATGGAGGAGATCCTCGCC	SSPCP expression
SSPCP-Rv	CCGCTCGAGGGTACGCCCGGCCAGGC	SSPCP expression
SPPT-Up-F	CCAAGCTT CCTGGCAGTAGTGTTGGTG	Integration vector
SPPT-Up-R	GGTAACGAAAACTAGTCGTACGAGGTCAGTTTAAACAGCG	Integration vector
SPPT-Dn-F	AAACTGACCTCGTACG ACTAGTTTCGTTACCTTGGGCCG	Integration vector
SPPT-Dn-R	GTGAATTC GGGCTACACCGTCGCTAC	Integration vector
Syn-APPT-F	TAAAGAGGTATATATTAATGTTGCAGCATACTTGG	Integration vector
Syn-APPT-R	CATAGCTGTTTCCTGTGTCAAAAAACCCCTCAAGACCCGTTTAGA GGCCCCAAGGGGTTATGCTAGTCAATAATGCCAGAATTTTG	Colony PCR
Syn-MPPT-F	TAAAGAGGTATATATTAATGTTTATATCTACCGATG	Colony PCR
Syn-MPPT-R	CATAGCTGTTTCCTGTGTCAAAAAACCCCTCAAGACCCGTTTAGA GGCCCCAAGGGGTTATGCTAGTCATAGATCAGAAAGGCC	Colony PCR
Syn-SFP-F	TAAAGAGGTATATATTAATGAAAATTTATGGGATTTAC	Colony PCR
Syn-SFP-R	CATAGCTGTTTCCTGTGTCAAAAAACCCCTCAAGACCCGTTTAGA GGCCCCAAGGGGTTATGCTAGCTACAACAGTTCTTCATAG	Colony PCR
Ptrc-F	CTCGTACGATTCTGAAATGAGCTGTTG	Colony PCR

Table S1. Continued

Primer	Sequence 5'-3'	Function
Ptrc-APPT-R	CCAAGTATGCTGCAACATTAATATATACCTCTTTA	Colony PCR
Ptrc-MPPT-R	CATCGGTAGATATAAACATTAATATATACCTCTTTA	Colony PCR
Ptrc-SFP-R	GTAAATCCCATAAATTTTCATTAATATATACCTCTTTA	Colony PCR
Kana-F	GTCTTGAGGGGTTTTTTG ACACAGGAAACAGCTATG	Colony PCR
Kana-R	AAACTAGTAAACGACGGCCAGTGAAT	Colony PCR
RT-rnpB-F	CGTGAGGACAGTGCCACAG	RT-PCR
RT-rnpB-R	CGCTCTTACCGCACCTTTG	RT-PCR
RT-SPPT-F	TTTGATTGGCTTAAGTAC	RT-PCR
RT-SPPT-R	AATGCTTCCTTCGCTGTC	RT-PCR
RT-APPT-F	ATCTAGTGACGAATTAGC	RT-PCR
RT-APPT-R	AATAAACCACTCTCGGC	RT-PCR
RT-MPPT-F	GTATTAACTATCAATTGC	RT-PCR
RT-MPPT-R	AAGCTATCTAAATCTTTC	RT-PCR
RT-Sfp-F	TAGTCATTCTGGTCGCTG	RT-PCR
RT-Sfp-R	ATAAATCAGAGTATTCGG	RT-PCR

Table S2. PPTases used in the phylogenetic analysis.

Accession Number	Gene name/locus tag	Organisms	Length (aa)
ABA22212.1	PPTase[a]	*Anabaena variabilis ATCC 29413*	237
WP_044522635.1	PPTase	*Nostoc sp. PCC 7120*	237
WP_004163140.1	PPTase	*Microcystis aeruginosa NIES-843*	220
ABB57835.1	HetI protein-like	*Synechococcus elongatus PCC 7942*	259
WP_012307697.1	PPTase	*Synechococcus sp. PCC 7002*	227
WP_010873553.1	PPTase	*Synechocystis sp. PCC 6803*	246
WP_017309026.1	PPTase	*Fischerella sp. PCC 9339*	240
ACG68433.1	Sfp	*Bacillus subtilis*	224
WP_009782852.1	PPTase	*Lyngbya sp. PCC 8106*	239
WP_026092908.1	PPTase	*Calothrix sp. PCC 7103*	241
WP_016950943.1	PPTase	*Anabaena sp. PCC 7108*	240
AAW67221.1	PPTase	*Nodularia spumigena NSOR10*	239
WP_015186867.1	PPTase	*Gloeocapsa sp. PCC 7428*	253
EHJ11403.1	PPTase	*Crocosphaera watsonii WH 0003*	248
WP_006529694.1	PPTase	*Gloeocapsa sp. PCC 73106*	242
WP_051044566.1	hypothetical protein	*Pleurocapsa sp. PCC 7319*	243
WP_006511535.1	PPTase	*Xenococcus sp. PCC 7305*	255
BAU66329.1	PPTase	*Stanieria sp. NIES-3757*	250
WP_017660318.1	hypothetical protein	*Geitlerinema sp. PCC 7105*	226
CUR17315.1	PPTase	*Planktothrix sp. PCC 11201*	249
WP_015112227.1	PPTase	*Nostoc sp. PCC 7107*	243
SCY12562.1	PPTase	*Nitrosospira sp. Nsp13*	246
WP_017306450.1	PPTase	*Spirulina subsalsa*	235
WP_029633554.1	hypothetical protein	*Scytonema hofmanni UTEX B 1581*	235
OCQ99688.1	PPTase	*Oscillatoriales cyanobacterium USR001*	254
WP_057178475.1	PPTase	*Cylindrospermopsis sp. CR12*	240
WP_041933312.1	PPTase	*Cyanothece sp. PCC 7822*	240
WP_054469188.1	hypothetical protein	*Planktothricoides sp. SR001*	247
WP_059000742.1	hypothetical protein	*Leptolyngbya sp. NIES-2104*	235
WP_015127290.1	PPTase	*Calothrix sp. PCC 7507*	234
ZP_00107102.1	PPTase	*Nostoc punctiforme PCC 73102*	239
ACN96032.1	holo-acyl-carrier-protein	*Fischerella sp. MV11*	128
WP_015181769.1	PPTase	*Microcoleus sp. PCC 7113*	139
AFY89096.1	PPTase	*Chroococcidiopsis thermalis PCC 7203*	137
WP_006519439.1	holo-ACP synthase	*Leptolyngbya sp. PCC 7375*	129
NP_926954.1	ACP synthase	*Gloeobacter violaceus PCC 7421*	132
AFY65439.1	holo-acyl-carrier-protein	*Geitlerinema sp. PCC 7407*	148
WP_006634204.1	holo-ACP synthase	*Microcoleus vaginatus*	157
WP_015187908.1	holo-ACP synthase	*Gloeocapsa sp. PCC 7428*	129
BAL39319.1	holo-acyl-carrier-protein	*Escherichia coli str. K-12 substr. MDS42*	126
AAH75207.1	MGC84206 protein	*Xenopus laevis*	302
XP_040785.1	PPTase	*Homo sapiens*	309
AGP54231.1	PPTase	*Streptomyces rapamycinicus NRRL 5491*	247

[a]: PPTase is an abbreviation of 4'-phosphopantetheinyl transferase.

Table S3. Selected CPs for the characterization of PPTases.

CPs	Strains	Metabolites	Biosynthetic pathway	Apo-form MW	Holo-form MW		GenBank accession number
					Calculated[d]	Observed	
SFACP	*Synechocystis* sp. PCC6083	Fatty acid	FAS	9655.69	9995.69	9997.78	ssl2084
AFACP	*Anabaena* sp. PCC7120	Fatty acid	FAS	10282.20	10622.20	10622.65	WP_010997493.1
APACP	*Anabaena* sp. PCC7120	Glycolipid	PKS	10804.09	11144.09	11012.84[a]	WP_010999481.1
APNPCP	*Anabaena* sp. PCC7120	Unknown	NRPS/PKS	12115.69	12455.69	12455.54	WP_010996791.1
FNPCP	*Fischerella* sp. PCC9339	Unknown	NRPS	10699.21	11039.21	10908.39[a]	WP_017308667.1
FisPCP	*Fischerella* sp. PCC9339	Shinorine	NRPS	12475.06	12815.06	12683.13[a]	WP_017312907.1
FNsACP	*Fischerella* sp. PCC9339	Unknown[b]	NRPS/PKS	15215.20	15555.20	15422.01[a]	WP_017308557.1
AprACP	*Moorea bouillonii*	Apratoxin	NRPS/PKS	10693.38	11033.38	11035.41	ctg1_8[c]
MACP	*Microcystis aeruginosa* NIES843	Unknown	NRPS/PKS	11106.54	11446.54	11314.81[a]	WP_012265828.1
ScACP	*Streptomyces coelicolor* A(3)2	Concanamycin	PKS	11186.50	11526.50	11526.67	WP_011030786.1
SsPCP	*Streptomyces scabiei* 87.22	Thaxtomin	PKS	8240.44	8580.44	8580.49	CBG75339.1

[a]Values corresponded to the proteins without the *N*-terminal methionine residue; [b]FNsACP is a homolog of NdaC from *Nodularia spumigena* NSOR10; [c]gene sequence is provided in the supporting information; [d]The MW of a holo-form CP was calculated by adding the mass of a phosphopantetheinyl moiety (340 Da) to that of its apo-form.

Table S4. Relative activity of seven PPTases in activating 11 CPs[a].

PPTase	CPs										
	**SFACP**	**AFACP**	**APNPCP**	**FNPCP**	**FisPCP**	**AprACP**	**APACP**	**MACP**	**FNsACP**	**ScACP**	**SsPCP**
APPT	99.1 ± 1.1	100 ± 2.0	100 ± 1.4	75.4 ± 2.9	100 ± 1.5	100 ± 1.4	100 ± 1.6	89.7 ± 3.9	100 ± 1.4	64.9 ± 3.1	28.8 ± 3.8
AvPPT	98.3 ± 2.4	98.7 ± 0.6	98.6 ± 2.6	81.9 ± 1.4	93.7 ± 3.2	93.7 ± 1.2	22 ± 2.3	88.5 ± 2.5	94.5 ± 0.6	28.6 ± 2.1	32.8 ± 2.7
MPPT	94.8 ± 1.3	99.6 ± 1.7	93.9 ± 1.3	96.2 ± 0.6	97.4 ± 3.6	93.2 ± 1.9	84.6 ± 3.8	100 ± 2.1	93.5 ± 1.3	18.8 ± 0.6	24 ± 3.9
FPPT	96.1 ± 2.1	94.5 ± 0.8	0.0	100 ± 0.7	78.5 ± 3.1	89.9 ± 2.1	10.1 ± 1.0	0.0	91.0 ± 2.4	0.0	29 ± 3.2
SePPT	99.5 ± 0.8	94.9 ± 2.2	0.0	30.5 ± 2.7	47.3 ± 2.8	11.9 ± 1.1	11.9 ± 0.8	0.0	43.6 ± 3.3	0.0	0.0
SPPT	100 ± 1.6	98.8 ± 3.1	71.6 ± 2.3	96.1 ± 1.5	87.8 ± 1.3	79.3 ± 2.7	79.3 ± 4.1	16.1 ± 1.7	79.2 ± 3.2	0.0	8.3 ± 0.8
Sfp	98.6 ± 1.7	96.2 ± 1.3	53.3 ± 3.8	82.8 ± 1.9	25.1 ± 1.5	99.6 ± 2.2	98 ± 3.3	97.1 ± 2.9	92.9 ± 2.0	100 ± 1.7	100 ± 1.0

[a]The data represented mean ± SD of three independent experiments.

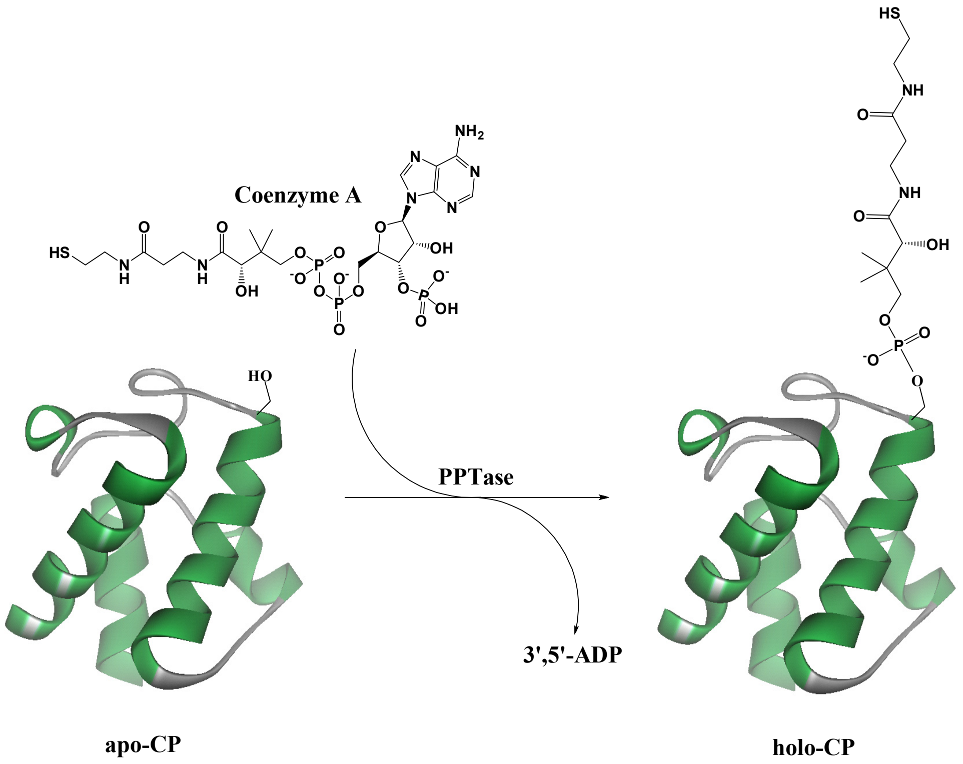

Fig. S1. Schematic representation of post-translational phosphopantetheinylation of a CP domain by a PPTase. The modification leads to a mass increase of 340 Dalton.

```
Sfp      ------------------------------------------MKIYGIYMDRPLSQEENE     18
SePPT    MQRPNPSDAVPVPSIPSCDRGPIPNPVTWRTSPEPLFLSAQTVHLWRCSLTRSLSSAE--     58
SPPT     --------------------------------------MLPQPQIWLCPTDRPL--IP--     18
MPPT     -----------------------------------MFISTDEVHLYFISLDPSGDRLE--     23
FPPT     --------------------MGSETNHLWLTAPTNLTLLPDDVHVWRISLDRPESELQ--     38
AvPPT    -----------------------MLQHTWLPKPPNLTLLSDEVHLWRIPLDRPESQLQ--     35
APPT     -----------------------MLQHTWLPKPPNLTLLSDEVHLWRIPLDQPESQLQ--     35

Sfp      RFMTFISPEKREKCRRFYHKEDAHRTLLGDVLVRSVISRQYQLDKSDIRFSTQEYGKPCI     78
SePPT    QAI---VAADCDRAQA-YGSNRRHQFLCGRWWLRQLLSLYLPEEPADFRFQLSPTGKPEL     114
SPPT     GYQALLSSEEMARGERYQRPQDKQRFLTMRLALRILLARQLDCLPQQLQFTYGPQGKPEL     78
MPPT     TLASLLSEDEIIRANRYHFPEHKRRFLVARGCLREILGSYLAISPEKIEFIYSERGKPSI     83
FPPT     ALQTTLSSDEIARAQRFYFEQHRQRFVAGRGILRTILGRYLGVEPQAVEFTYELRGKPLL     98
AvPPT    HLAATLSSDELARANRFYFPEHRQRFTAGRGILRSILGLYLGVEPKQVKFEYESRGKPVL     95
APPT     DLAATLSSDELARANRFYFPEHRRRFTAGRGILRSILGGYLGVEPGQVKFDYESRGKPIL     95

                                       * *
Sfp      PDL---PDAHFNISHSGRWVIGAFD-SQPIGIDIEKTKPI--SLEIAKRFFSKTEYSDLL     132
SePPT    PQ----SNLCFNLSHSGSTLLIAIAW-QPVGVDVEQPRSR-SWLALARRYFPSAELAAMQ     168
SPPT     VDRERR-SPWFNVAHSGNYGLIGLSTEGEIGVDLQIMLPKPHYLKLAKRFFAPQEVQQLE     137
MPPT     NY-----QLQFNLSHSEEMAICGLTLTARIGVDLEKMRQMKDLDSLTKRFFCAREHELVE     138
FPPT     ADRFADSGVSFNLSHSQDLALCGVSRNRKIGIDVEYMRSVSDVEALAERFFAPREYEVVR     158
AvPPT    GDRFADSGLLFNLSHSQNLGLCAVNYTRQIGIDLEYLRPTSDLESLAKRFFLPREYELLR     155
APPT     GDRFAESGLLFNLSHSQNLALCAVNYTRQIGIDLEYLRPTSDLESLAKRFFLPREYELLR     155

                           *
Sfp      AKDKDEQTDYFYHLWSMKESFIKQEGKGLSLPLDSFSVRLHQDGQVSIELPDSHSPCYIK     192
SePPT    Q--STDCDRWGLASWVCKEAWIKAQGRTLANSLRHLQCAWTANGQPRLSGLGSEES-QVQ     225
SPPT     SLEGEKRTKLFYQLWTAKEAFLKATGKGISGGLNQVIPDENLAKYQYLPDS----G-DTN     192
MPPT     K--SAEKEKLFFQLWTAKEAYLKAVGTGISGGLDRVEVGLNPLKLD--NVA----G-EWQ     189
FPPT     SLPSNQQQQVFFRYWTCKEAYLKAIGVGIVQ-LEKVEISLTLEQPAKLITD----E-EWS     212
AvPPT    SLPDEQKQKIFFRYWTCKEAYLKATGDGIAK-LEEIEIALTPTEPAKLQTT----P-AWS     209
APPT     SLPDEQKQKIFFRYWTCKEAYLKATGDGIAK-LEEIEIALTPTEPAKLQTA----P-AWS     209

Sfp      TYEVDPGYK--MAVCAAHPDFPEDIT---MVSYEELL-----------------------     224
SePPT    LLQVDPQEQLWAAI-AMPAGWNYQTWTAAIIRKNH-------------------------     259
SPPT     HWRLSSQ------PLLADQGSNDNYWMAIAWCTNEVNQVESNYLPNIQPFQWPRNLDSLP     246
MPPT     LWTAAIGDNYRATVVIEGSDRVIKTF-----GLSDL------------------------     220
FPPT     LIELVPGDHYLGAVAIAGQNLDLKYW-----QY---------------------------     240
AvPPT    LLELVPDDNCVAAVAVAGFGWQPKFW-----QY---------------------------     237
APPT     LLELVPDDNCVAAVAVAGFGWQPKFW-----HY---------------------------     237
```

Fig. S2. Multiple-sequence alignment of characterized cyanobacterial PPTases and Sfp. The completely conserved residues are shaded in gray. The proposed magnesium binding residues are indicated with asterisks (*). Boxed region indicates the conserved W/KEA motif.

SFACP

ATCAGGAAATTTTTGAAAAAGTAAAAAAAATCGTCGTGGAACAGTTGGAAGTGGATCCTGACAAAGTGA
CCCCCGATGCCACCTTTGCCGAAGATTTAGGGGCTGATTCCCTCGATACAGTGGAATTGGTCATGGCCCT
GGAAGAAGAGTTTGATATTGAAATTCCCGATGAAGTGGCGGAAACCATTGATACCGTGGGCAAAGCCGT
TGAGCATATCGAAAGTAAA

AFACP

GCCAATCAGAAACTTTTGAAAAAGTCAAAAAAATTGTTATCGAACAACTAAGTGTGGAGAACCCTGACA
CAGTAACTCCAGAAGCTAGTTTTGCCAACGATTTACAGGCTGATTCCCTCGATACAGTAGAACTAGTAAT
GGCTTTGGAAGAAGAATTTGATATCGAAATTCCCGATGAAGCCGCAGAGAAAATTACCACTGTTCAAGA
AGCGGTGGATTACATCAATAACCAAGTTGCCGCATCAGCT

APACP

GTCTAAAACAAAATTATAGTGCAGCAGATATTCAAGCTTGGATGATATCTAATCTAGCTGAATTGTTGGG
AGTAGATGGTGATGAAATCGATGCTACTGTCAATTTAGAAAGCTATGGTTTGGATTCGGCACAGGCAAT
GGTACTAGTTAGTAAACTAGAGCAATTGTTGGGATTTCAACCATCACCTTTGTTGTTGTGGCATTACCCC
ACTATTGAATCGTTGTCTGAACGTTTAGCTGAAGAATTGGAAGAACAATCT

APNPCP

AACAATCTACAACTAATCACGCCCGCCCCCAAATTACCGCTACCTACCTTCCCCCCAGCAATGAAATTGA
AGCCAGAGTCACCCAAGTAATGGAGAGTTTATTGGGAATCGCTCCTATTGGGGTTAATGATAACTTCTTT
GAGTTAGGAGGACATTCCCTGTTAGCAATTCAAGCAGTTTCACAGCTACGGGAAGAATTTCAAGTAGAA
TTACCCATGCGACAATTTTTATTTGAGTCACCCACAATTGGGGGGATAGCCAAAATTATCATTGAAAATC
AATCGCCTATTACTGAT

FNPCP

CCCAACGCCCTATCATTATCCCTCGTACAAATACTGAACAGCGAATAGGCGAGATTTGGAAGAAGGCGA
TGAAGTGGGATTCTGTCTCGATATGTGATGATTTCTTTGAATCTGGCGGAAATTCACTTATTGCTGTGAG
AATAATCAACGCTATCAACAAAGAATTTCATTGTGCCTTGCCTTTACATGCTCTTTTTGAAGCTCCAAGC
ATTGAAAAGCTCGCTCATAAGGTTGATAGTGATGAAGTTGAA

FNsACP

GCTTTTCTAGAAGATGTCCCTCCAACAGAACGTCGAGAACACTTATTAGAATATCTTGGAAAAGAAGTA
GCAAAAATCTTAGGAATAAAACATATACCCGACCCAGAACAAGGATTTATAGAAATGGGAATTGACTCT
TTGCTTTCCATTGAATTCAAAAATCGTTTAGAAAAAGGATTAGAAATTGCTTTACCATCTACTTTAATATT
TGATTTTCCGAATATTAGCAAATTAAATAATTATCTATTTGAGCAAATTTATGGTTGGGAAGTAAATACT
ACCGTGGAGACAACTGTTGATATTGTAGAAGTTAATGAAGATTTAATTTTGCAAGAACTGGCAGATTTA
GAAGCTTTTCTAGGTAATTCCCTCGAGCACCACCACCACCACCACTGA

FisPCP

AGGCGATCGCTTCCCAAACCTGATTTTTCTAACTTAATCACTCATGAAGATTTTACGCCTGCACGCAATG
ATTTAGAGAGAAAAATCGCGCAGATTTGGTCAGAAATTTTACAGATTTCGGAAATTGATATTAGAGATA
ACTTTTTTGAAGTTGGTGGTAATTCCCTTTTAGCATTACATTTAATGAATGCCATCGAACAAAAATTTGGT
CGAGAGTTAGCACTGTCAACTTTACTTACTAATAACTCAATTGAAAAACTAGCAGAAATTCTGCAAAAC
CCCACAGATGTTTTTCCCAATTCA

AprACP

AAATTTTTGAACAGGAATGTCGAAAATTATTAAAATCTCTACTGGGTGTTCAACGTATGGAGAGATTGCC
TGGTGACACACCACTAATGGAGTCAGGAATGGATTCACTGGAGTTGTTAGAATTTCGTGCTCTTATAGAA
AGAAAGTTTGGGATTAAGTTAAAGTCTACCTTCTTTTTAGTTACAAAACTCTTATAGCGGTAGCAGAGT
ATCTTTCAGAACGGGAAGATATTAATTTTAGT

MACP

TGACAACTGTTCAATCTCCTTGTACCGTTGAAGACATTCAAAACTGGCTCGTTGATCAGTTTGCTCAACA
ACTCGATGTTGACCTTGATGACATTGATATTGAAGAACCTTTTGATAATTATGAACTCGACTCACGAAAA
GCGTTAGTTTTATTAGGACGCTTAGAAAAATGGCTCGGAAAGGAATTAAATCCTGTGGTCATTTTTAACT
ATCCCACCATTGCTGAATTAGCAACCCGATTAGGGGAATTATATCTT

ScACP

GAGCAGCGGCTGGCTCCGCTGTCCGCGGCCGAGCGCGAGCGGGCACTCACGGATCTCGTGCGCGTCCAG
GTCGCGGCGGTGCTCGGGCACTCTGACCCCGGCGCGATCGAGTCCGGCCGGGCCTTCCAGGAGCTGGGC
TTCGACTCACTGACAGCCGTCGAACTTCGCAACCAGCTGAGCACCGCGAGCGGACTGCGCCTGCCCACC
ACCCTCGTCTTCGACCACCCCTCCCCCGCCGCTCTCGCCGCCCACCTCTCGGCGGAGCTGTTCGGCGAGC
AGGAG

SsPCP

GCCCGCCGGCTCGAACCGTTGGACGAACCCGCGCGACGCCGTCTGCTGCTCGACCTGGTGTGCGACCAC
GCGGCCGCGGTCCTCGGCCACACCGGCCGCCAGGCCGTCCCGGCCGACCAGGCGTTCTCCGCCGTCGGG
TTCGACTCGATGCTCGCCGTGTCCTTCCGTAACCGGCTGCGCACCGCGACCGGCGTCCCCGTCGCCGCGA
CGGTGGTGTTCGACCATCCCACCCCCGCCGCCCTCGCCGACCACCTGTACGACGGGTTGAGCGCCCGTCC
CGGACCGGCCGTT

Codon optimized *Sfp*

ATGAAAATTTATGGGATTTACATGGATAGACCCCTGAGCCAAGAAGAAAACGAACGCTTTATGACCTTT
ATTAGCCCTGAAAAACGGGAAAAATGTCGCCGTTTTTATCATAAAGAAGATGCCCATCGTACCTTATTGG
GTGATGTGTTGGTTCGGAGTGTGATTTCTCGCCAATACCAATTGGATAAAAGTGATATTCGGTTTTCTAC
TCAAGAATATGGTAAACCCTGTATTCCCGATTTGCCCGATGCCCATTTTAATATTAGTCATTCTGGTCGCT
GGGTTATTGGTGCTTTTGATAGTCAACCCATTGGTATTGATATTGAAAAAACCAAACCCATTTCTTTGGA
AATTGCCAAACGCTTTTTCAGTAAAACCGAATACTCTGATTTATTGGCTAAAGATAAAGATGAACAAACT
GATTACTTTTACCATTTGTGGAGTATGAAAGAATCTTTTATTAAACAAGAAGGTAAAGGTTTAAGTTTGC
CCTTAGATAGTTTTTCTGTGCGGTTGCATCAAGATGGTCAAGTTAGTATTGAATTACCCGATAGTCATTCT
CCCTGTTACATTAAAACTTATGAAGTTGATCCCGGTTATAAAATGGCTGTTTGTGCAGCACACCCCGATT
TTCCAGAAGATATTACTATGGTTTCCTATGAAGAACTGTTGTAG

Fig. S3. Nucleotide sequences of selected wild type CP genes and codon optimized Sfp gene.

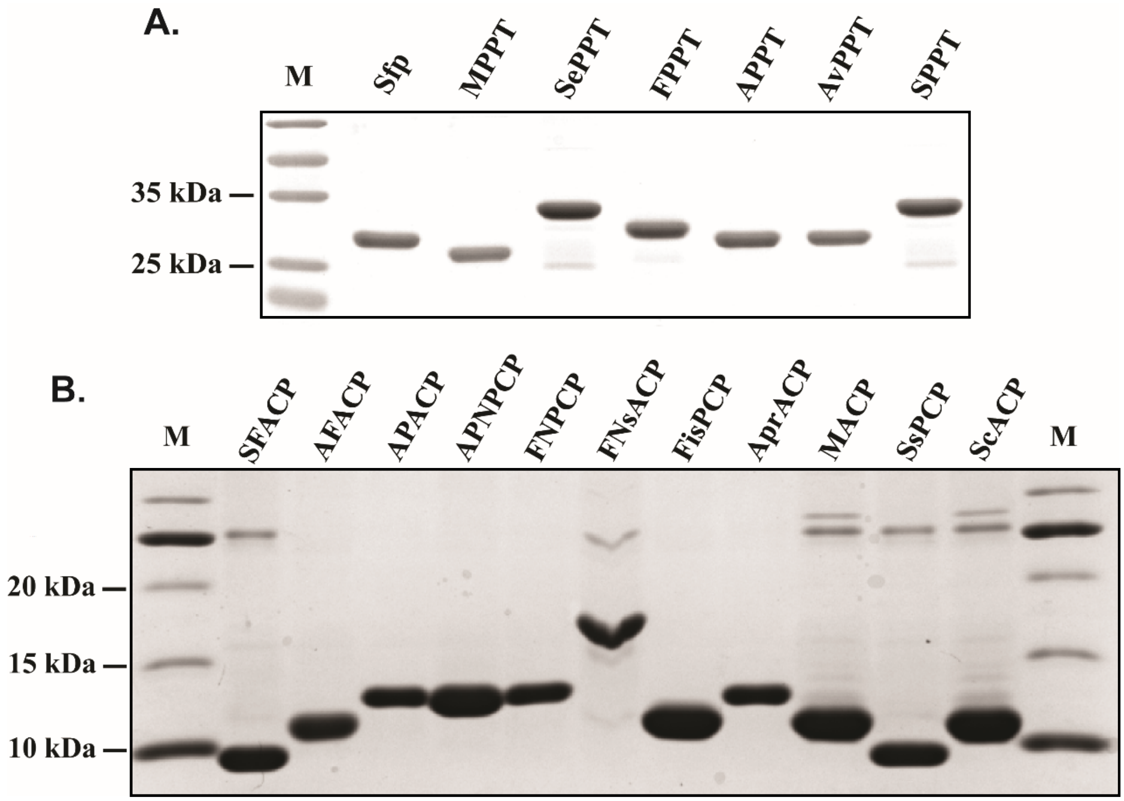

Fig. S4. SDS-PAGE (15%) analysis of the purified PPTase (A) and CP (B) proteins. All proteins showed expected molecular weights and CPs were validated in LC-MS analysis.

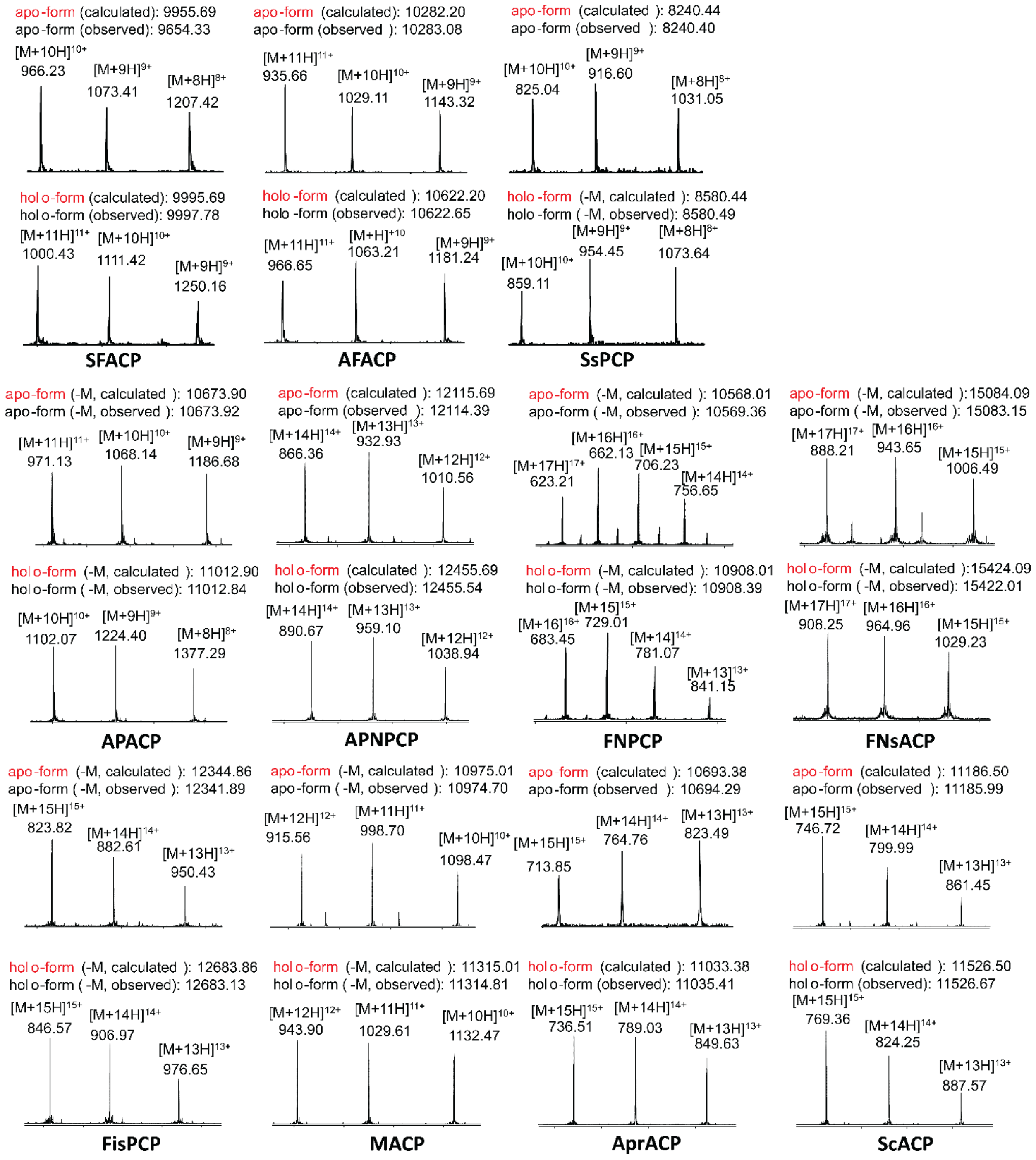

Fig. S5. HR-MS spectra of apo- and holo-CPs. The charge status, *m*/*z* value, and calculated and observed molecular weights of CPs were shown.

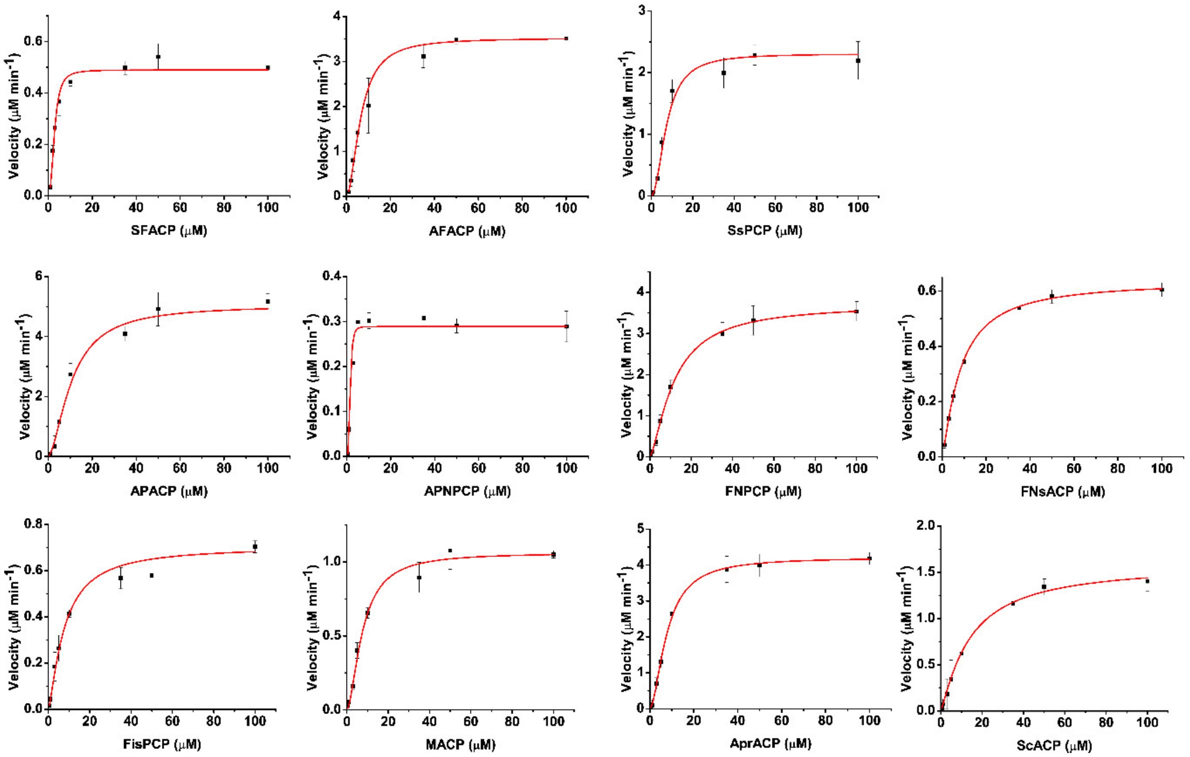

Fig. S6. Michaelis-Menten kinetic analysis of APPT evaluated for 11 selected CP substrates.

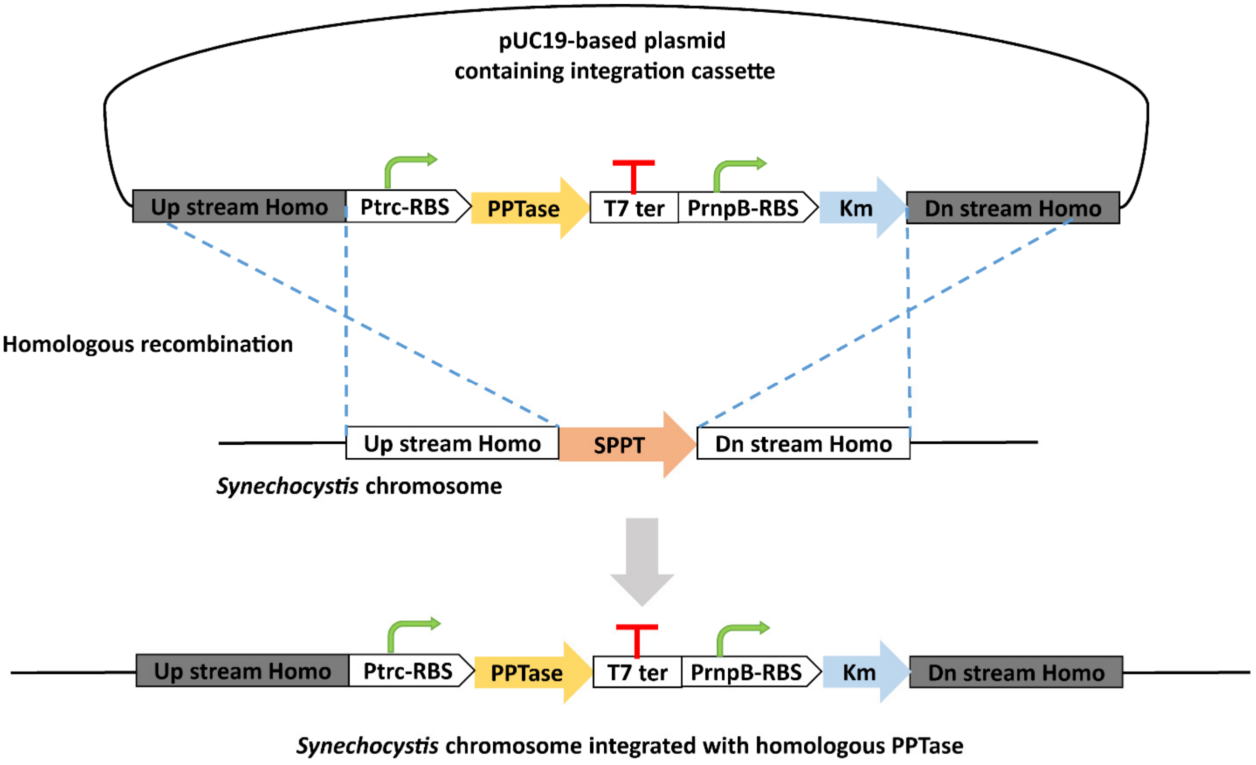

Fig. S7. Schematic representation of homologous replacement of the *SPPT* gene with foreign *PPTase* genes in *Synechocystis*.

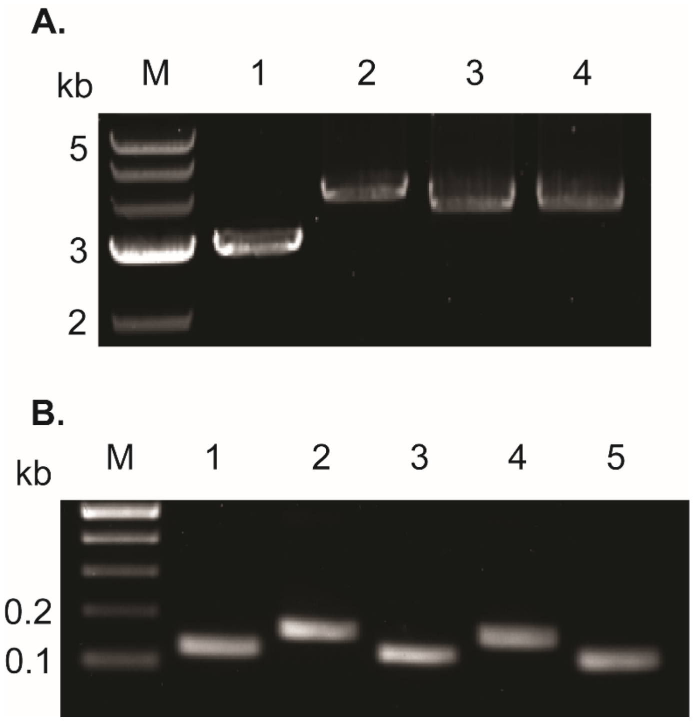

Fig. S8. PCR analyses of the integration and transcription of the *APPT*, *MPPT* and *Sfp* genes in *Synechocystis* mutants. (**A**) PCR diagnosis of *Synechocystis* mutants whose *SPPT* gene was chromosomally replaced by the *APPT*, *MPPT* and *Sfp* genes. The *SPPT* gene was detected in the wild type (lane 1) but not in three mutants (lanes 2 to 4). The *APPT*, *MPPT* and *Sfp* genes were found in one of three mutants, respectively (lanes 2 to 4). (**B**) RT-PCR analysis of the transcription of *SPPT*, *APPT*, *MPPT* and *Sfp* genes in *Synechocystis* wild type and mutants (lanes 2 to 5, respectively). The *rnpB* gene was used as a positive control (lane 1).
